# Study on the Application of the Combination of TMD Simulation and Umbrella Sampling in PMF Calculation for Molecular Conformational Transitions

**DOI:** 10.3390/ijms17050692

**Published:** 2016-05-09

**Authors:** Qing Wang, Tuo Xue, Chunnian Song, Yan Wang, Guangju Chen

**Affiliations:** Key Laboratory of Theoretical and Computational Photochemistry, Ministry of Education, College of Chemistry, Beijing Normal University, Beijing 100875, China; wangqing0409@163.com (Q.W.); xuetuogo@126.com (T.X.); scn@mail.bnu.edu.cn (C.S.)

**Keywords:** potential of mean force (PMF) calculations, CMD/TMD simulations, umbrella sampling, dihedral rotation, unfolding transition

## Abstract

Free energy calculations of the potential of mean force (PMF) based on the combination of targeted molecular dynamics (TMD) simulations and umbrella samplings as a function of physical coordinates have been applied to explore the detailed pathways and the corresponding free energy profiles for the conformational transition processes of the butane molecule and the 35-residue villin headpiece subdomain (HP35). The accurate PMF profiles for describing the dihedral rotation of butane under both coordinates of dihedral rotation and root mean square deviation (RMSD) variation were obtained based on the different umbrella samplings from the same TMD simulations. The initial structures for the umbrella samplings can be conveniently selected from the TMD trajectories. For the application of this computational method in the unfolding process of the HP35 protein, the PMF calculation along with the coordinate of the radius of gyration (*R*_g_) presents the gradual increase of free energies by about 1 kcal/mol with the energy fluctuations. The feature of conformational transition for the unfolding process of the HP35 protein shows that the spherical structure extends and the middle α-helix unfolds firstly, followed by the unfolding of other α-helices. The computational method for the PMF calculations based on the combination of TMD simulations and umbrella samplings provided a valuable strategy in investigating detailed conformational transition pathways for other allosteric processes.

## 1. Introduction

It is well known that the characterization of the structure and the energetics of allostery for biological systems play a crucial role in understanding biological functions of cell signaling and metabolism regulation [1,2,3,4,5]. People not only require the structural information of conformational changes in biological systems, but also are greatly interested in the energetical information during the conformational changes. However, the previous experiments on detailed conformational variations and energetical information for the conformational changes at the atomic level are very limited so far. Therefore, theoretical calculation of free energies is of great importance for understanding the kinetics and the structural determinants of biomolecular processes [6]. Free energy calculations based on molecular dynamics (MD) simulations provide useful tools for obtaining the energy information of conformational transitions. Currently, the thermodynamic integration method, the perturbation method, and the potential of mean force (PMF) method are widely used in calculating the free energy changes for the biologic processes of macromolecular systems [7,8,9,10,11]. The PMF, first introduced by Kirkwood in 1935 [12], provides a 1D free energy map along a defined reaction coordinate for describing characters of biomolecular systems in modern statistical mechanical theories of liquids [13].

Various theoretical studies on PMF calculations of biologic processes, such as conformational transition and base-pair flipping of DNA, folding and unfolding of proteins, transports of protons and small molecules through channels, and ligand-binding to receptors, *etc.*, have made many gratifying achievements in recent years [14,15,16,17,18,19,20,21,22,23,24,25,26,27,28,29,30,31,32,33,34,35,36,37,38]. It has been reported that the PMF calculations provided the energetical changes of the flipping of the Watson–Crick (WC) paired C and G bases in the DNA dodecamer, and the aggregation of two Dickerson–Drew B-DNA molecules in the presence of polyethylenimine molecules along with the reaction coordinates of center-of-mass (COM) dihedral and distance [39,40]. The energetics of K^+^, Cl^−^, and Ca^2+^ ions passing through the gramicidin A channel and the K^+^ channel from *Streptomyces lividans* under the distances of mass center of two ions were revealed using PMF calculations [41,42]. The small root mean square deviation (RMSD)-PMF calculations for searching the local energy minima of ligands were also performed [43,44,45,46,47,48]. Variations of PMF as a function of physical coordinates depend on the pathway chosen for the transition. In most cases, it is difficult to search the pathway of a complex conformational transition due to existence of distinct possible pathways. It is well known that the algorithm of targeted molecular dynamics (TMD) offered a convenient tool to search for a possible pathway of transitions between two conformations, and the free energy calculations along the pathway provided a method for the approximation of the free energy barrier of the conformation transition. Therefore, PMF calculations based on the TMD simulations have been applied to investigate some conformational transitions of biologic systems [49,50,51]. The PMF profiles were obtained for the pathways of B–A and B–Z transitions in DNA searched by the TMD simulations [52,53,54]. Zhan and co-workers reported the detailed pathways and free energy profile for a structural transformation from a nonprereactive complex to a prereactive one (BChE-(−)-cocaine) associated with the (−)-cocaine rotation in the binding pocket by using the combined TMD and PMF simulations [55]. Schatz and co-workers studied the PMF profile on the ordered and disordered transition of 90 peptide amphiphiles (Pas) along with the coordinate of the radius of gyration (*R*_g_) based on the TMD simulation [56]. Especially, the free energy variations of the conformational transition process of the N′ to N conformations of the alternate frame folding (AFF) calbindin-D_9k_ protein in Ca^2+^-free form were investigated by PMF calculation and umbrella samplings in our recent work [57]. To the best of our knowledge, the applications of PMF calculation based on TMD simulations on the conformational transition process in bio-systems have not yet been widely adopted. Moreover, there is a disappointing lack of in-depth discussion in the computational method combining both TMD simulations and umbrella samplings for accurate PMF calculations. The detailed discussion of such method for calculating possible pathways and the accurate free energy variations will be valuable for activating the research field of large-scale conformational transitions of biologic systems.

In order to introduce the computational method of PMF calculations based on the combination of TMD simulations and umbrella samplings, we carried out PMF calculations for the conformational transitions of a small molecule and a protein along different coordinates. Our main objectives were (1) to address the accuracy of PMF calculations for a rotation transition process of a typical butane molecule based on the same TMD simulation and different umbrella samplings under two transition coordinates of dihedral rotation and RMSD variation; (2) to investigate feasibility of the combination of TMD simulation and umbrella samplings to select initial structures of sampling simulations; (3) to explore the application of this method on the unfolding transition of the 35-residue subdomain of the villin headpiece (HP35) [58,59].

## 2. Results and Discussion

Geometry optimizations for a butane molecule were computed at the B3LYP/6-31++G(d,p) [60,61] levels of theory using the Gaussian 09 program [62]. The structural parameters of butane are shown in Table S1 of Supplementary Materials with the experimental data [63,64,65]. Similarly, some conformations of butane with the different dihedral rotations of C–C–C–C were also optimized. For the conventional molecular dynamics (CMD) and targeted molecular dynamics (TMD) simulations of the butane molecule, all atom types were generated using the ANTECHAMBER module in the AMBER 9 program [66]. Each of the conformations was explicitly solvated by using the TIP3P water potential inside an orthorhombic box of water molecules with a minimum solute-wall distance of 8 Å.

Based on the previous experimental studies, the folded structure of the 35-residue subdomain of the villin headpiece (HP35) was chosen from the crystal structure of HP35 protein (PDB: 1YRF) with the amino acid sequence of LSDEDFKAVFGMTRSAFANLPLWKQQHLKKEKGLF, and is assigned as HP35-R model [58]. The initial coordinates of the full-unfolded structure of HP35, assigned as HP35-L model, were constructed using the LEAP module in the AMBER 9 program. The Cartesian coordinates from the crystal structure of sequence of HP35 protein and the full-unfolded HP35 sequence respectively for the initial structures of two HP35-R and HP35-L models were employed for the CMD and TMD simulations.

The potential of mean force (PMF) with umbrella samplings is a useful method to describe the free energy change associated with conformational transition processes of molecular and biological systems. The determination of initial structures of umbrella samplings along the coordinates of conformational transition is crucial to calculate PMF curve. TMD simulation can observe large-scale conformational transition between two known end-point conformations of a molecule, and provides a useful tool to obtain initial structures for further umbrella sampling simulations. Therefore, based on the combination of TMD simulation and umbrella samplings, PMF calculations along a conformational transition coordinate can be achieved by using the weighed histogram analysis method (WHAM).

### 2.1. Potential of Mean Force (PMF) Calculations Based on the Combination of Targeted Molecular Dynamics (TMD) Simulation and Umbrella Samplings along Different Coordinates for the Dihedral Rotation of Butane

In order to explore accuracy of PMF calculations for the conformational transition, the PMF calculations for the dihedral rotation of butane, as an example, were performed by using TMD simulation and umbrella samplings along both coordinates of dihedral rotation and RMSD variation.

#### 2.1.1. Targeted Molecular Dynamics Simulations for Rotations of Butane

**Figure 1:**
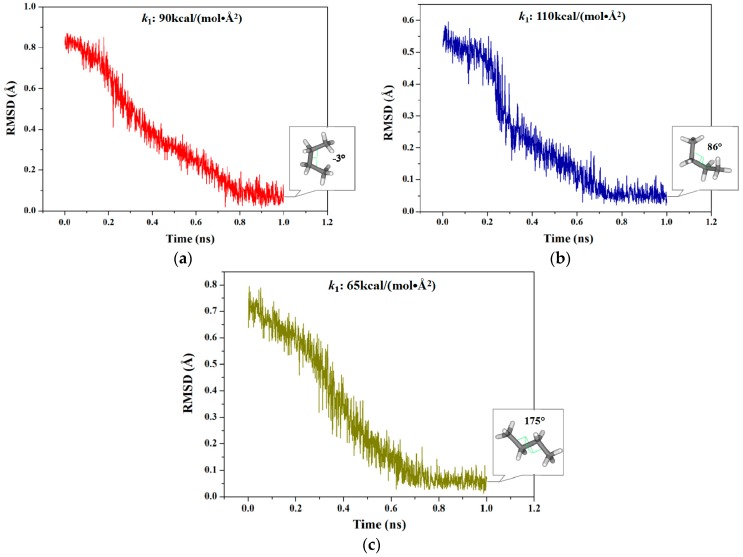
Root mean square deviation (RMSD) values of four C atoms of butane from three individual targeted molecular dynamics (TMD) simulations corresponding to three dihedral angle sections with the respective appropriate force constants: (**a**) 0.84→0.00 Å (−180°→0°) in red; (**b**) 0.53→0.00 Å (−60°→90°) in blue; and (**c**) 0.73→0.00 Å (60°→180°) in dark yellow. The terminal structure and the corresponding dihedral angle value for each TMD simulation are marked in the square.

Due to the TMD simulation based on the RMSD values as the offset parameter, a corresponding range of RMSD caused by the certain dihedral rotations of C–C–C–C can be firstly detected. However, RMSD could not describe the deviation between two conformations with a complete periodic rotation of 360°. Thus, three TMD simulations for the dihedral rotation from −180° to 180° for a butane molecule were performed with the biasing potentials along the RMSD variation by using AMBER 9 program. Such three TMD simulations correspond to three sections of dihedral variations of −180°~0°, −60°~90° and 60°~180° with certain overlaps due to this periodic limitation of dihedral angle. Since TMD simulation is used for determining the transition pathways between two known conformations, two dynamically stable states of structures were obtained by CMD simulations based on the optimized conformations for each of the TMD simulations. In the first section (−180°→0°), the calculated range of RMSD is from 0.84 to 0.00 Å referenced from the starting structure. For the other two sections (−60°→90° and 60°→180°), such RMSD values were calculated from 0.53 and 0.73 Å to 0.00 Å, respectively. To obtain the appropriate force constant for the first section, three independent-short (1 ns) TMD simulations were performed using different force constants of 50, 70 and 90 kcal/(mol·Å^2^), respectively. As shown in Figure 1a for this section, the simulation at the appropriate force constant of 90 kcal/(mol·Å^2^) reached the RMSD value of <0.15 Å and the closest targeted dihedral value of −3° for the targeted structure extracting from the C–C–C–C atoms of butane in the trajectory. However, the simulations under other two force constants of 50 and 70 kcal/(mol·Å^2^) did not reach the expected targeted dihedral value. Similarly, Figure 1b,c show that the other two simulations under the appropriate force constants of 110 kcal/(mol·Å^2^) and 65 kcal/(mol·Å^2^) reached the RMSD values of <0.15 Å and the closest targeted dihedral values of 86° and 175°, respectively. Therefore, such three TMD simulations were then used to determine the initial structures for the further umbrella samplings and to complete the PMF calculations for the whole rotational transition process of butane.

#### 2.1.2. PMF Calculation along with the Coordinate of Dihedral Angle

**Figure 2:**
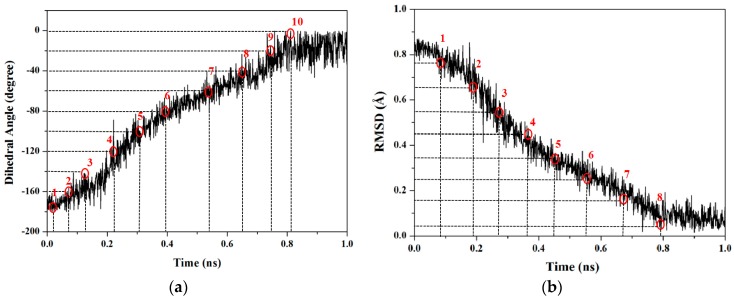
(**a**) The dihedral angle values of C–C–C–C and the 10 selected sampling windows; and (**b**) the root mean square deviation (RMSD) values of four C atoms and the 8 selected sampling windows from the first TMD simulation. The selected sampling windows are numbered around the red circles. The corresponding expected values and snapshots with the red circles are marked by the dash lines.

**Figure 3:**
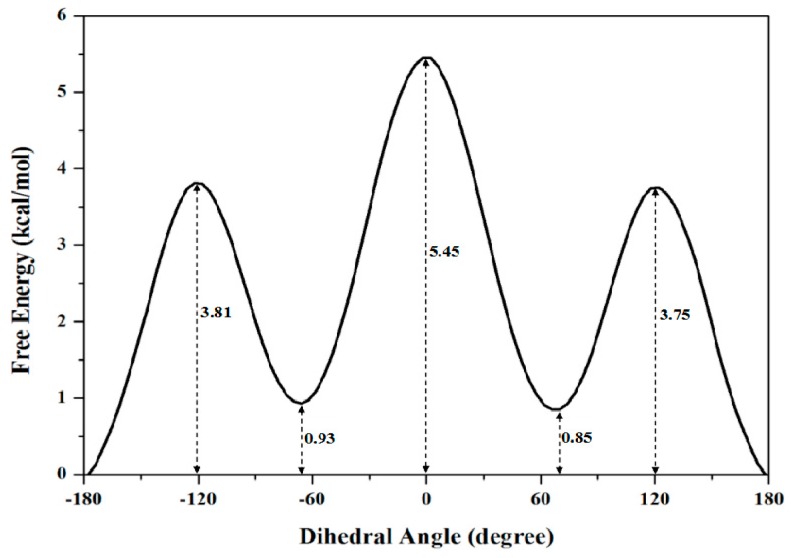
The calculated PMF profile for the rotational transition of butane from −180° to 180° with the relative energy values along the coordinate of dihedral angles. The numbers around the dash lines are the relative energy values at the corresponding angles.

To obtain the accurate PMF curve with the combination of TMD simulation and umbrella samplings, selection of the valid transition coordinate in a conformational transition process is necessary for the calculations of energetical changes. A valid transition coordinate should satisfy the simple linear variation feature to describe the transition process. To test the simple dihedral rotation as the valid coordinate, the variations of dihedral angles were extracted from the three TMD trajectories using the PTRAJ module of the AMBER 9 program. The corresponding results present the linear feature along with the simulation times, and are shown in Figure 2a for the first TMD simulation and Figure S1 of Supplementary Materials for other two simulations. Thus, the umbrella sampling windows can be selected along with the valid dihedral coordinate based on three TMD simulations. It can be seen from Figure 2a that the 10 windows of umbrella samplings for the first simulation were determined from the range of dihedral angles of −180° to 0° with 20° intervals. Similarly, the 4 and 5 windows of umbrella samplings for the second and third TMD simulations were respectively determined from the ranges of dihedral angles of 20° and 100° to 80° and 180° with same intervals (see Figure S1). Furthermore, the 10 snapshots at 0.02, 0.08, 0.13, 0.22, 0.31, 0.39, 0.55, 0.75 and 0.81 ns from the first TMD trajectory corresponding to these 10 windows can be considered as the initial structures for the further umbrella sampling simulations (see Figure 2a). The similar method was applied to the other two simulations (see Figure S1). Therefore, the total 19 umbrella sampling simulations with the corresponding initial structures and the harmonic potentials are applied to the 19 sampling windows to cross the energy ranges for the dihedral rotation of butane. Namely, a 500-ps MD simulation at 300 K for each sampling window was carried out with the appropriate restraint force constant of 33 kcal/(mol·rad^2^) referenced from the previous work [67]. The 19 output data recording the time development of the dihedral angle variations around the individual expected dihedral angle values were obtained, and were checked by Histogram multi program. Since all of the individual histograms present smooth, no major shift from expected, and no bare patch, 19 umbrella sampling simulations were proved to be sufficient enough. (See Figure S2 in Supplementary Materials) Due to the requirement of monotone floating expected values in the WHAM program for PMF calculation, the 19 output data from the sum of all sampling windows were used for further PMF calculations of the periodic rotation of 360° by using the WHAM analysis. The calculated PMF profile along the dihedral coordinates was gained, and is shown in Figure 3. The free energy variation of the dihedral rotation from −180° to 180° involves three energy tops and two energy minima. The free energies change from 0.00 kcal/mol at dihedral −178° with the anti-conformation to the first energy top of 3.81 kcal/mol at dihedral −120°, then to the first energy minimum of 0.93 kcal/mol at dihedral −66°, then to the highest top of 5.45 kcal/mol at dihedral 0° with the syn conformation, then to the second minimum of 0.85 kcal/mol at dihedral 66° with the gauche conformation, and to the third top of 3.75 kcal/mol at dihedral 120°, finally to 0.00 kcal/mol at dihedral 178° back to the anti-conformation. That is, such periodic transition of C–C–C–C dihedral angles of −180° to 180° involves two symmetric half-periodic transitions of dihedral angles of −180° to 0° and 0° to 180°. The first-half-periodic transition of −180° to 0° could occur with the barriers of 3.81 kcal/mol and 4.52 kcal/mol via an intermediate. Then, the energy variation of the second-half-periodic transition of 0° to 180° reproduces the reverse process of the first-half-periodic transition due to the symmetry torsion in butane. The current results shows that the PMF calculation with the combination of individual TMD simulations and umbrella samplings provided an accurate description of the whole periodic dihedral transition in a butane molecule.

#### 2.1.3. PMF Calculation along with the Coordinate of Root Mean Square Deviation (RMSD)

**Figure 4:**
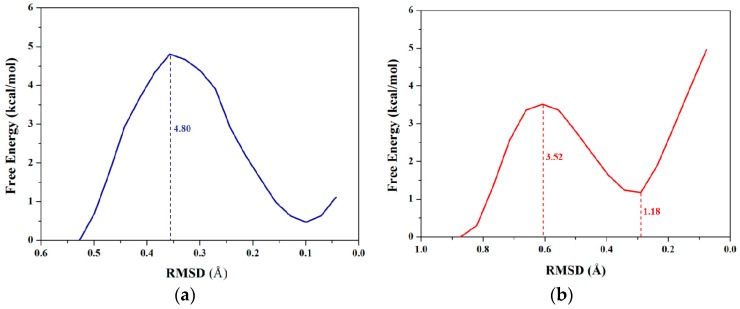

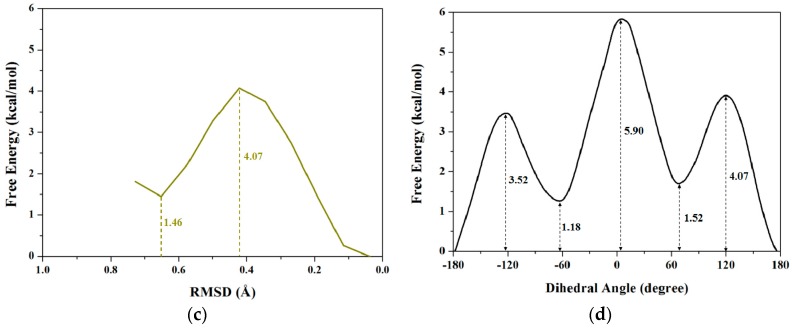
Three calculated PMF profiles along the coordinate of RMSD for three individual TMD simulations corresponding to three dihedral angle sections of butane with the relative energy values: (**a**) 0.84→0.00 Å in red; (**b**) 0.53→0.00 Å in blue; and (**c**) 0.73→0.00 Å in dark yellow; (**d**) The whole fitted PMF profile with the relative energy values along the periodic dihedral coordinate of −180°→180°. The dihedral angle values for the fitted profile were extracted from the RMSD values in three individual TMD simulations. The numbers around the dash lines are the relative energy values at the corresponding RMSDs and dihedral angles.

RMSD values could be used to describe conformational changes between two known end-point conformations along the TMD trajectory due to RMSD values served as the offset parameter of TMD simulation. Therefore, RMSD could be selected as a valid transition coordinate with the linear trend involving many degrees of freedom for conformational transition of a molecule (see Figure 1). In this work, we also applied such coordinate of RMSD for the dihedral rotation of butane to calculate the PMF profile based on the combination of TMD simulations and umbrella samplings. The umbrella sampling windows along with the RMSD coordinate can be selected from three TMD simulations discussed above. It can be seen from Figure 2b that the 8 windows of umbrella samplings for the first simulation were determined from the range of RMSDs of 0.84 to 0.04 Å with 0.1 Å intervals. The 8 snapshots at 0.09, 0.19, 0.28, 0.37, 0.45, 0.55, 0.68 and 0.79 ns from the first TMD trajectory corresponding to these 8 windows can be considered as the initial structures for the further umbrella sampling simulations (see Figure 2b). Similarly, the 5 and 7 windows of umbrella samplings for the second and third TMD simulations were respectively determined from the ranges of RMSDs of 0.53 and 0.73 to 0.01 and 0.04 Å with same intervals (see Figure S3 in Supplementary Materials). For umbrella sampling simulations, due to the force constants needed to restrain the sampling structure at the expected value in each window, the appropriate biasing force constants were tested, and used for all windows of three TMD simulations. Namely, for the first TMD simulation, the appropriate force constants applied in the umbrella sampling simulations of 8 windows range from 50 to 90 kcal/(mol·Å^2^). For other two TMD simulations, such appropriate force constants applied in those of 5 and 7 windows range from 80 and 50 to 160 and 90 kcal/(mol·Å^2^), respectively. A 500-ps MD simulation at 300 K for each of sampling windows was carried out with the corresponding initial structures from the TMD simulations, and appropriate force constants. Due to the requirement of monotone floating expected values in the WHAM program for PMF calculations, three individual PMF calculations along the respective RMSD coordinate (that describes the partial dihedral rotation) based on the three TMD simulations and the respective sampling windows could completely describe the whole dihedral rotation of butane, which is different from one PMF calculation along the dihedral coordinate of 360° rotation. For the first TMD simulation, the 8 output data recording the time development of RMSD variations around the individual expected RMSD values were obtained, and used for the corresponding PMF calculation along the RMSD coordinate of 0.84 to 0.00 Å. Similarly, for the second and third TMD simulations, the 5 and 7 output data were obtained, and also used for the second and third PMF calculations along the RMSD coordinates of 0.53 and 0.73 to 0.00 Å, respectively. The three corresponding PMF profiles are shown in Figure 4a–c, respectively. As shown in Figure 4a, the free energy variation presents the energy change from 0.00 kcal/mol at the RMSD value of 0.84 Å to the energy top of 3.52 kcal/mol at 0.61 Å, then to the energy minimum of 1.18 kcal/mol at 0.29 Å. Due to a specific RMSD value corresponding to a certain dihedral value in a structure, such PMF profile indicates that the RMSD values of 0.61 and 0.29 Å at the current first energy top and minimum match the dihedral angle values of −120° and −65.6° which is well consistent with those in the PMF profile along the dihedral coordinate discussed above. For Figure 4b, the free energies change from 0.00 kcal/mol at the RMSD value of 0.73 Å to the energy top of 4.80 kcal/mol at 0.36 Å, then to the energy minimum of 0.48 kcal/mol at 0.10 Å. In addition, for Figure 4c, the free energies change from the energy minimum of 1.46 kcal/mol at 0.65 Å to the top of 4.07 kcal/mol at 0.42 Å, then to 0.00 kcal/mol at 0.04 Å. To obtain the whole PMF profile representing the whole dihedral rotation of −180°→180°, three individual PMF profile can be integrated. To overcome the integrated deviation, the overlaps of boundaries in three individual PMF profiles were modified by using the method of adjacent averaging. The whole fitted PMF profile along the periodic dihedral coordinate of −180°→180° was obtained, and is depicted in Figure 4d. It can be seen that the fitted PMF profile presents the high consistence with that along the dihedral coordinate (see Figure 3), *i.e.*, the free energy change from 0 kcal/mol at the dihedral value of −179° to the first energy top of 3.52 kcal/mol at −120°, to the first energy minimum of 1.18 kcal/mol at −66°, to the second energy top of 5.90 kcal/mol at 1°, then to the second energy minimum of 1.52 kcal/mol at 68°, to the third energy top of 4.07 kcal/mol at 117°, finally back to 0.00 kcal/mol at dihedral 176°. To compare the accuracy of the PMF calculations, the stationary point energies for the half-periodic dihedral transitions of 0° to 180° along both coordinates of the dihedral rotation and RMSD variation are shown in Table 1 along with the previous calculated data. Table 1 predicts the small deviations of the relative free energies resulting from the different calculated models and water environments, *i.e.*, the PMF calculations based on the combination of TMD simulations and umbrella samplings with the explicit TIP3P water box (the current work), the PMF calculations based on CMD simulations and umbrella samplings with the implicit generalized Born solvent model, and the ab initio G2 calculation in vacuum. As expected, the same PMF profiles along with both conformational transition coordinates of dihedral rotation and RMSD variation can be accurately obtained by using the same TMD simulations and different umbrella samplings for the rotation process of butane.

### 2.2. PMF Calculation Based on the Combination of TMD Simulation and Umbrella Samplings for the Unfolding Process of 35-Residue Villin Headpiece Subdomain (HP35) Protein

#### 2.2.1. Targeted Molecular Dynamics Simulation for the Unfolding Process

**Figure 5:**
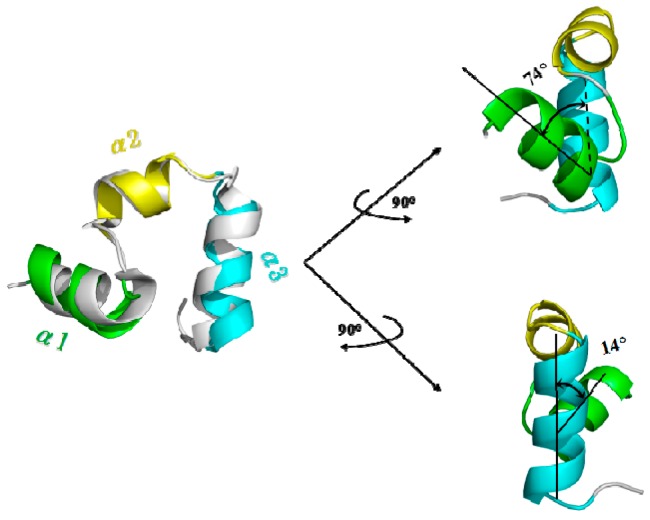
Simulated average structure for HP35-R model viewed from different directions with the superposition for the simulated structure and the crystal one in gray (PDB: 1YRF) on the left. The three helices of α1, α2 and α3 for the simulated structure are colored in green, yellow and cyan, respectively. The interhelical angle value between α1 and α2 helices, and the dihedral angle value between the planes of α1–α2 and α2–α3 are given.

To explore PMF calculation for complicated conformational transition of biological system, we selected the unfolding process for the benchmark protein, the 35-residue villin headpiece subdomain (HP35) (PDB: 1YRF), to calculate the PMF profile by using the combination of TMD simulations and umbrella samplings. To address the conformational transition process, we performed the TMD simulation from the folded HP35-R model to the unfolded HP35-L model at 340 K due to its experimentally melting temperature of 342 K [69,70,71]. We first carried out the CMD simulations on the folded HP35-R and unfolded HP35-L models to gain the stable structures of the two HP35 states for the further TMD simulations. The RMSD values of all the backbone atoms relative to the starting structure over the trajectory of CMD simulation at 340 K for the HP35-R model were examined to determine the system equilibrium. The corresponding plot of RMSD values over simulation times is shown in Figure S4 of Supplementary Materials, and presents that the folded HP35-R state reached equilibrium with the average RMSD value of <2.5 Å, which indicated that the simulated result of HP35-R state reproduced the crystal structure of HP35 protein (PDB: 1YRF). The 6-ns CMD trajectory analysis yielded the HP35-R equilibrated conformation (shown in Figure 5 with the superposition for the simulated structure and the crystal one) between 5 and 6 ns simulation times, recording 500 snapshots at every 2 ps time-interval of trajectory. The structure of HP35-R model includes three α-helices with α1 helix of Ser2~Gly11, α2 helix of Thr13~Pro20 and α3 helix of Leu21~Gly33. The interhelical angle of 74° between α1 and α2 helices using the program INTERHLX and the dihedral angle of 14° between the planes of α1–α2 and α2–α3 are also shown in Figure 5, which are consistent with the measured results of 95° and 4°, respectively, from the X-ray experimental results with the non-plane structure of three α helices. However, the full-unfolded HP35-L model was only simulated for 500 ps due to the instability of its loop structure, and the corresponding conformation has also been yielded. Based on the dynamically stable states of the HP35-R and HP35-L models, we successfully gained the folding→unfolding transition process from the started HP35-R model to HP35-L model using 6-ns TMD simulation at 340 K with the appropriate biasing force constant of 3.0 kcal/(mol·Å^2^) applied onto all the backbone atoms. The corresponding transition process involves the deformation of tertiary structure of protein from the spherical structure to the linear one firstly, then the unfolding of secondary structure of three α helices. Therefore, such TMD simulation was then used to determine the initial structures for the further umbrella samplings and the PMF calculations for the unfolding process of HP35.

#### 2.2.2. PMF Calculation along with the Coordinate of the Radius of Gyration

**Figure 6:**
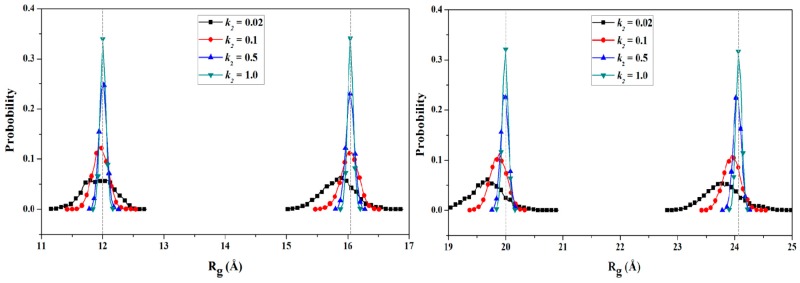
Probability distribution of the radius of gyration (*R*_g_) for four representative umbrella sampling simulations centered at *R*_g_ = 12.0, 16.0, 20.0, 24.0 Å for four selected force constants of 0.02, 0.1, 0.5, 1.0 kcal/(mol·Å^2^), respectively.

**Figure 7:**
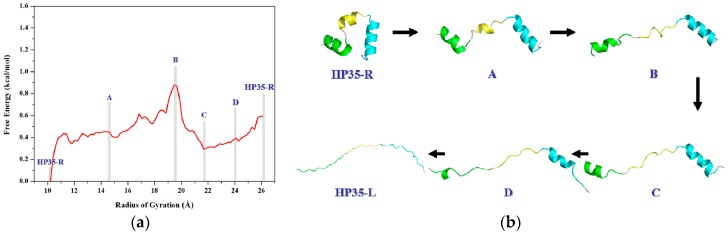
(**a**) Calculated free energy profile for the transition process of HP35-R to HP35-L along with the reaction coordinate of the radius of gyration (*R*_g_). The mazarine letters denote the different conformations; (**b**) The three-dimensional average structures for the six corresponding conformations of HP35-R, A, B, C, D and HP35-L presented in (**a**). The three helices of α1, α2 and α3 for the simulated structure are colored in green, yellow and cyan, respectively.

To obtain the PMF profile with the combination of TMD simulation and umbrella samplings, selection of the valid coordinate for the conformational transition process of protein is necessary. Based on the general applied coordinates for the protein allosteries, the radius of gyration (*R*_g_) was selected as the valid transition coordinate for the PMF calculation of such unfolding process of HP35 due to its simple linear variation feature and widespread application for folding↔unfolding transitions of proteins. The variations of *R*_g_ values were also extracted from the 6-ns TMD trajectory using the PTRAJ module of the AMBER 9 program. 82 windows of umbrella samplings from the TMD simulation were determined from the range of the Cα radius of gyration values (*R*_g_) of 10.2 to 26.4 Å with 0.2 Å intervals. Furthermore, the 82 snapshots from the TMD trajectory corresponding to these windows can be considered as the initial structures for the further umbrella sampling simulations. For umbrella sampling simulations, four restraint force constants of 0.02, 0.1, 0.5 and 1.0 kcal/(mol·Å^2^) were selected to apply respectively to the tested four windows of *R*_g_ = 12.0, 16.0, 20.0, 24.0 Å. The probability distributions of *R*_g_ under four different restraint force constants at four tested-expected *R*_g_ values are shown in Figure 6. The probability distribution with *k*_2_ = 1.0 kcal/(mol·Å^2^) possesses a narrow single peak at each tested-expected *R*_g_ value, which predicts the appropriate force constant of *k_2_* = 1.0 kcal/(mol·Å^2^) for further umbrella sampling simulations for the transition of HP35-R to HP35-L. A 2-ns CMD sampling simulation at 340 K for each of sampling windows was carried out with the corresponding initial structures extracted from the TMD simulation, and with the appropriate force constant. The 82 output data recording the time development around the individual expected values were also checked by Histogram_multi program. Based on the 82 output data and the expected *R*_g_ values, the PMF calculation along the *R*_g_ coordinates of the unfolding process of HP35 was performed by using the WHAM program, and is depicted in Figure 7a. According to free energy changes along the coordinate of *R*_g_, the conformational transition from the reactant of HP35-R to the product of HP35-L mainly involves two transition steps: one is from the reactant to the top energy state with the free energy increase of 0.90 kcal/mol; the other is from the top energy state to the product with the certain energetical fluctuations. Based on the conformational analysis for the transition process, four transition conformations of A, B, C and D were extracted from the trajectory. The corresponding time-average conformations and two models of HP35-R and HP35-L from the CMD simulations are shown in Figure 7b.

**Figure 8:**
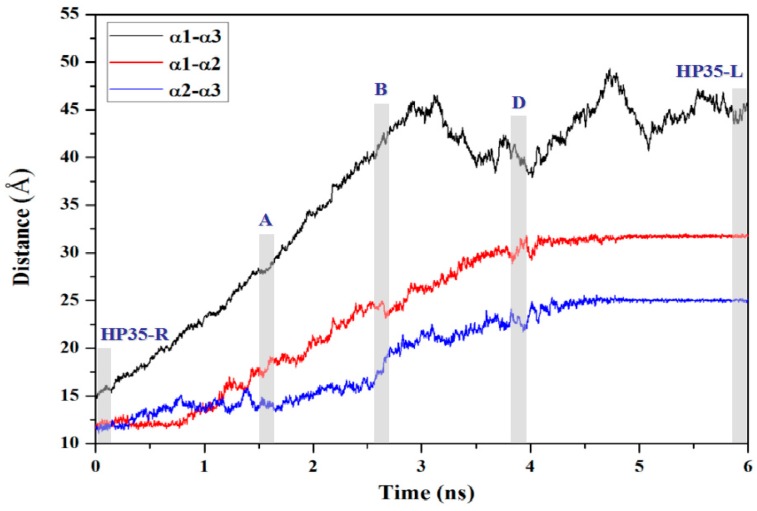
Mass center distances between α1 and α3 helices (black),between α1 and α2 helices (red), and between α2 and α3 helices (blue) along the transition pathway from the simulation of HP35-R to HP35-L; the mazarine letters in the grey areas denote HP35-R, A, B, C, D and HP35-L conformations.

**Figure 9:**
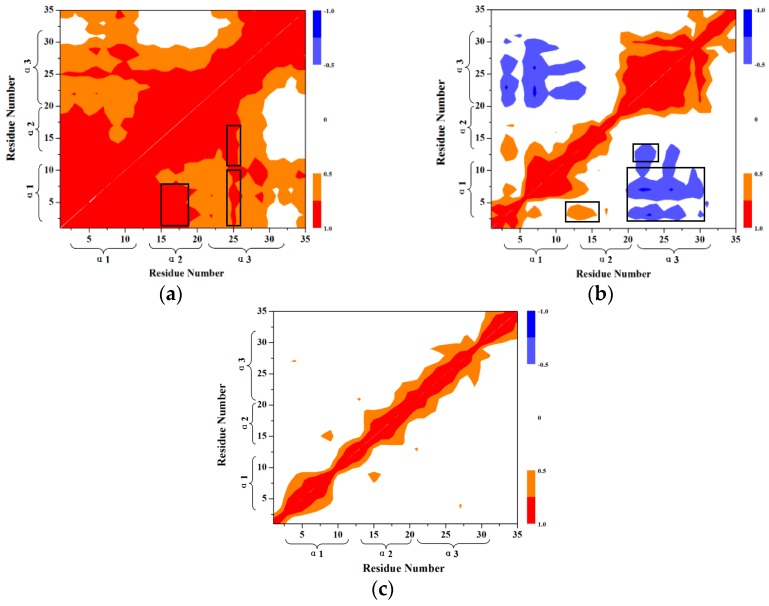
Dynamical cross-correlation maps for (**a**) HP35-R model; (**b**) HP35-L model; (**c**) B conformation with the key sub-regions squared in black.

To further interpret the transition conformations from the HP35-R to HP35-L models, the analyses of the mass center distances between the two adjacent α helices, and the intra-helix hydrogen bonds were quantitatively performed with the PTRAJ module of the AMBER 9 program and are shown in Figure 8 and Table 2, respectively. In the first step from the HP35-R reactant to A, then to B conformations, the free energies gradually increase from 0.0 kcal/mol at HP35-R to 0.45 kcal/mol at A, then to the energy top of 0.90 kcal/mol at B, which is consistent with the previous experimental results of ~1 kcal/mol at 340 K and the theoretical data of ~2 kcal/mol [59,71,72,73,74]. The structural changes involve that the spherical structure of three α helices at the HP35-R reactant changes to the deformational tertiary structure with the extending of the α1 and α3 helices far from each other, and with the slightly unfolding of the middle α2 helix at A. Then, at B, the α1 and α3 helices continually extend with the further unfolding of the α2 helix. These results are supported by the gradual increases of the mass center distances between the α1 and α3 helices from 14.7 Å at the HP35-R reactant to 28.9 Å at A, then to 41.7 Å at B. Simultaneously, the mass center distances between the α1 and α2 helices, and between the α2 and α3 helices also increase from 11.7 and 11.8 Å at the HP35-R reactant to 18.9 and 13.9 Å at A, then to 23.7 and 18.9 Å at B, respectively (see Figure 8). The deformation of tertiary structure involving mainly in the extending of the α1 and α3 helices spends the significant free energy requirement of 0.90 kcal/mol at the energy top. On the other hand, the relative percentages of the total hydrogen bond occupancies in the extended α2 helix decrease from 20.96% at the HP35-R reactant to 7.01% at B due to the unfolding of the α2 helix. (The computational details of the relative percentages of the total hydrogen bond occupancies were given in Section S2 of Supplementary Materials) For example, the hydrogen bond between the N–H group of Phe17 and the O atom of Thr13 in the α2 helix is maintained with the occupancy of 99.32% of simulation times in the HP35-R reactant; while it disappears in B (see Table 1). In the second step from B to C, and to D conformations, then to the HP35-L product, the structural changes involve that the deformational tertiary structure B changes to the further deformational structure C with the small energy fluctuation by 0.59 kcal/mol. Then, at D, the unfolding of α1 helix causes the energy fluctuation of 0.10 kcal/mol with the decrease of the relative percentages of the total hydrogen bond occupancies in the extended α1 helix from 11.11% at B to 2.75% at D. For example, the hydrogen bond between the N–H group of Asp5 and the O atom of Leu1 in the α1 helix is maintained with the occupancies of 99.72% of simulation times in B; while it disappears in D (see Table 1). Finally, at the HP35-L product, the α3 helix occurs the full unfolding with the energy fluctuation of 0.21 kcal/mol and with the disappearing of the hydrogen bonds in the unfolding α3 helix at HP35-L from the 23.04% relative occupancy percentage at D. The HP35-L product presents the full extended-linear structure with the endo-thermodynamic energy of 0.60 kcal/mol referencing to HP35-R reactant. It is worth pointing out that the first unfolding of the middle α2 helix play the crucial mediation for the unfolding transition process of HP35 protein. To explore the conformational communication of each residue along the transition pathway of the HP35-R reactant to the HP35-L product, we constructed and analyzed the motion correlations for all Cα atoms from the simulation trajectories. The motion correlations for the HP35-R model, HP35-L model and B conformation are displayed in Figure 9a–c, respectively. The results show that the motion correlations between the residues range from high correlations (red) to high anticorrelations (blue). These maps show the high motion correlations between the residues. As expected, from Figure 9a, the motions of three α helices of α1, α2, and α3 in HP35-R reactant significantly correlate with each other represented by the black squares due to the spherical structure with the tight adjacency of three α helices. The large correlated motions of the α1 helix with the α3 helix involve the correlation communication of the middle α2 helix. In the B conformation in Figure 9b, such correlations of three α1, α2, and α3 helices decrease; and the correlations between the α1 and α3 helices, and between the α2 and α3 helices become slight anticorrelations, compared to those in the HP35-R reactant, due to the extending of the spherical tertiary structure and the movement of the α1 and α3 helices far away from each other. This result predicts the transition characteristics for the B conformation in the transition process of the HP35-R reactant to the HP35-L product. However, in the HP35-L product in Figure 9c, such correlations of three α1, α2, and α3 helices are insignificant, compared to those in the HP35-R reactant, due to the linear tertiary structure with three helices far away from each other. Summarily, the transferring of the correlations among α1, α2 and α3 helices from HP35-R reactant to B conformation, then to HP35-L product further illustrates the mediation feature of α2 helix during this unfolding transition process.

As expected, the PMF calculation along the *R*_g_ coordinate with the combination of TMD simulation and umbrella samplings could efficiently describe the free energy change of unfolding process of a protein. The PMF calculations based on the combination of TMD simulations and umbrella samplings may be valuable in computational studies of detailed pathways and free energy profiles for other similar conformational transitions.

## 3. Models and Methods

### 3.1. Conventional Molecular Dynamics Simulation

All conventional molecular dynamics (CMD) simulations for these systems were carried out using the AMBER 9 package [66] and ff03 all atom force field parameters [75,76,77]. 2 Cl^−^ ions were used to neutralize each of the HP35-R and HP35-L models, and an ionic strength of 350 mM was generated by adding 88 Na^+^ and 88 Cl^−^ ions [58]. Each of the models was similarly solvated by using the TIP3P water potential with a minimum distance of 8 Å. The computational details of the CMD procedure are given in Section S1 of Supplementary Materials.

### 3.2. Targeted Molecular Dynamics Simulation

Targeted molecular dynamics (TMD) simulation is a method to observe large-scale conformational transition between two known end-point conformations of a molecule. A restraint energy term was added to the energy function proportional to the square of the difference which may be characterized as the mass-weighted RMSD of the current structure to the target structure in terms of atomic positions [78,79]. The functional form of the restraint energy can be written as:

(1) $$E_{TMD}=\frac{1}{2}k_{1}N{\left[RMSD(t)-RMSD_{0}(t)\right]}^{2}$$

where, *k*_1_ is the harmonic force constant per atom, *N* is the number of the restrained atoms, *RMSD*(*t*) is the root mean square deviation of the simulated structure at time *t* relative to the target structure, and *RMSD*_0_(*t*) is the prescribed target RMSD value at time *t* that decreases to zero linearly with time to drive the system from a starting structure to the target structure. Various *k*_1_ values for the TMD simulations need to be tested to determine which one is appropriate for all of the systems concerned in the present study as the harmonic force constant applied onto all the backbone atoms to bias the trajectories toward the target structure.

### 3.3. Umbrella Sampling and the Potential of Mean Force

In order to explore the free energy profile for the conformational transition, PMF was calculated by using umbrella sampling MD simulations and the weighed histogram analysis method (WHAM) [13,80,81,82,83]. Based on the characteristics of transition processes for different systems, some convenient transition coordinates were tested to satisfy the simple linear variation features in order to effectively describe the whole transition processes. In umbrella sampling calculations, the biasing harmonic potentials were introduced to constrain each conformation to a narrow range of reaction coordinates. The functional form of the restraining potential for reaction coordinate *r* for the *i*th window in the current umbrella sampling was:

(2) $$V_{i}(r)=\frac{1}{2}k_{2}{\left(r_{i}-r_{i0}\right)}^{2}$$

where *k*_2_ is the force constant and *r_i_*_0_is the center of the window. Some windows from the selected coordinate were generated for the transition processes. The analysis of all the trajectories, and the calculations of the geometric parameters of the molecule and protein were performed using the PTRAJ module of the AMBER 9 program. After all the umbrella-sampling MD simulations were finished, the data collected from separate simulation windows were combined along the reaction coordinate. These data were then used to calculate the PMF profile for the whole structural transition process with the WHAM using the code developed by Alan Grossfield [81,84].

### 3.4. Calculations of Interhelical Angle and Correlation of Atomic Motions

To analyze conformational changes in the relative orientations of any two helices, the program INTERHLX [85] was used. The dynamic feature of a protein and the extent of correlation of motions in the different regions of a protein were assessed via the calculation of cross-correlation coefficients, *C*(*i*,*j*), given as follows:

(3) $$C(i,j)=\langle \Delta r_{i}\times \Delta r_{j}\rangle /{\left(\langle \Delta r_{i}^{2}\rangle \langle \Delta r_{j}^{2}\rangle \right)}^{\frac{1}{2}}$$

In the equation, ∆*r_i_* and ∆*r_j_* are the displacement vectors for Cα atoms of residues *i* and *j*, respectively, and the angle brackets denote the ensemble averages [66,86]. The computational details of the INTERHLX procedure and the motion correlations are given in Section S2 of Supplementary Materials.

## 4. Conclusions

The computational method for the PMF calculations based on the combination of TMD simulations and umbrella samplings has been introduced for the conformational transition processes for a butane molecule and the protein of 35-residue villin headpiece subdomain (HP35) by using AMBER 9 program and the weighed histogram analysis method (WHAM). It is demonstrated in this method that the TMD simulation can provide the obvious images of large-scale conformational transitions that are applied to selection of initial structures of umbrella samplings for PMF calculations. Three individual TMD simulations for the dihedral rotation of a butane molecule from −180° to 180° were performed with the biasing potentials due to the limitation of RMSD used to describing a complete periodic rotation of 360°. By using the combination of TMD simulations and umbrella samplings, the PMF calculations under both coordinates of dihedral rotation and RMSD variation for a butane molecule were performed. For the dihedral coordinate of −180° to 180°, based on the umbrella sampling windows selected from three individual TMD simulations, the 19 sampling data from the sum of all sampling windows were used for the PMF calculation. The PMF profile demonstrated that the free energy changes involve three energy tops and two energy minima at the dihedral angles of −120°, 0° and 120°, and at −66° and 66°, respectively. For the RMSD coordinate, three individual PMF calculations along the respective RMSD coordinate based on the same TMD simulations and the respective sampling windows were performed for the dihedral rotation of −180° to 180° of butane. The free energy variations including three energy tops and two energy minima are involved in the three individual PMF profiles along the RMSD coordinates. Then, the modified PMF profile representing the whole dihedral rotation of −180°→180° can be obtained through integrating such three individual PMF profiles. It can be predicted that the free energy changes for a dihedral rotation process can be accurately described under different conformational transition coordinates of dihedral rotation and RMSD variation. To investigate the application of this computational approach in large-scale conformational transition, the PMF calculation along with the coordinate of the radius of gyration (*R*_g_) for the unfolding process of the HP35 protein was carried out. The PMF profile of the transition process from the folded HP35-R reactant with the spherical structure of three α helices to the unfolded HP35-L product with a linear structure presents the gradual increase of free energies by about 1 kcal/mol with the energy fluctuations. The structural transition from the folded HP35-R reactant to the unfolded HP35-L product involves the extending of the spherical structure firstly, followed by the unfolding of three α helices with the mediation feature of α2 helix. It can be demonstrated that the computational method for the PMF calculations based on the combination of TMD simulations and umbrella samplings provided a valuable strategy in investigating detailed conformational transition pathways for other similar allosteric problems.

## Figures and Tables

**Table 1:** Comparisons of free energies of stationary point structures.

Coordinates	Free Energy (kcal/mol)			
	anti	120°	gauche	syn
**Dihedral Angle**	0	3.75	0.85	5.45
**RMSD**	0	4.07	1.52	5.9
**References**	0 ^a^	4.22 ^a^	1.44 ^a^	5.86 ^a^
	0 ^b^	3.83 ^b^	0.66 ^b^	6.48 ^b^

^a^ Taken from Ref. [67]; ^b^ Taken from Ref. [68].

**Table 2:** Occupancies (%) of hydrogen bonds for the HP35-R, B, D and HP35-L conformations.

Helices	Hydrogen Bond	HP35-R	B	D	HP35-L
**α1**	(Ala8)N–H···O(Glu4)	95.32	–	–	–
	(Val9)N–H···O(Asp5)	99.96	–	–	–
	(Asp5)N–H···O(Leu1)	99.56	99.72	–	–
	(Phe6)N–H···O(Ser2)	95.56	97.20	–	–
	(Lys7)N–H···O(Glu4)	97.92	69.64	65.92	–
	(Phe10)N–H···O(Phe6)	99.72	–	–	–
	(Glu4)N–H···O(Leu1)	67.40	–	–	–
	(Gly11)N–H···O(Lys7)	52.60	–	–	–
**α2**	(Ala18)N–H···O(Agr14)	99.76	99.68	–	–
	(Leu20)N–H···O(Phe17)	97.48	–	–	–
	(Phe17)N–H···O(Thr13)	99.32	–	–	–
	(Asn19)N–H···O(Ala16)	71.00	–	–	–
	(Asn19)N–H···O(Ser15)	71.80	68.56	–	–
	(Ala16)N–H···O(Thr13)	63.76	–	–	–
**α3**	(Gln26)N–H···O(Leu22)	100	99.92	99.28	–
	(Leu34)N–H···O(Lys29)	98.88	99.80	–	–
	(Glu31)N–H···O(Hie27)	99.48	98.88	98.40	–
	(Lys32)N–H···O(Leu28)	99.72	99.88	–	–
	(Lys30)N–H···O(Gln26)	99.80	87.48	90.08	–
	(Gly33)N–H···O(Lys30)	91.88	88.64	–	–
	(Lys29)N–H···O(Gln25)	99.92	98.88	–	–
	(Leu28)N–H···O(Lys24)	98.68	96.56	99.84	–
	(Hie27)N–H···O(Trp23)	96.44	84.12	80.80	–
	(Gly25)N–H···O(Leu22)	96.24	54.12	84.60	–

## Supplementary Materials

Supplementary materials can be found at http://www.mdpi.com/1422-0067/17/5/692/s1.

## References

[1] Brandsdal B.O., Österberg F., Almlöf M., Feierberg I., Luzhkov V.B., Åqvist J. (2003). Free energy calculations and ligand binding. Adv. Protein Chem..

[2] Fenton A.W. (2008). Allostery: An illustrated definition for the ‘second secret of life’. Trends Biochem. Sci..

[3] Hilbert M., Kebbel F., Gubaev A., Klostermeier D. (2011). eIF4G stimulates the activity of the DEAD box protein eIF4A by a conformational guidance mechanism. Nucleic Acids Res..

[4] Kenakin T., Miller L.J. (2010). Seven transmembrane receptors as shapeshifting proteins: The impact of allosteric modulation and functional selectivity on new drug discovery. Pharmacol. Rev..

[5] Luo X., Tang Z., Xia G., Wassmann K., Matsumoto T., Rizo J., Yu H. (2004). The Mad2 spindle checkpoint protein has two distinct natively folded states. Nat. Struct. Mol. Biol..

[6] Park S., Khalili-Araghi F., Tajkhorshid E., Schulten K. (2003). Free energy calculation from steered molecular dynamics simulations using Jarzynski’s equality. J. Chem. Phys..

[7] Hénin J., Chipot C. (2004). Overcoming free energy barriers using unconstrained molecular dynamics simulations. J. Chem. Phys..

[8] Kofke D.A. (2005). Free energy methods in molecular simulation. Fluid Phase Equilib..

[9] Pearlman D.A. (1994). A comparison of alternative approaches to free energy calculations. J. Phys. Chem..

[10] Smith D.E., Haymet A. (1993). Free energy, entropy, and internal energy of hydrophobic interactions: Computer simulations. J. Chem. Phys..

[11] Tobias D.J., Brooks C.L. (1987). Calculation of free energy surfaces using the methods of thermodynamic perturbation theory. Chem. Phys. Lett..

[12] Kirkwood J.G. (1935). Statistical mechanics of fluid mixtures. J. Chem. Phys..

[13] Roux B. (1995). The calculation of the potential of mean force using computer simulations. Comput. Phys. Commun..

[14] Allen T.W., Andersen O.S., Roux B. (2006). Molecular dynamics—Potential of mean force calculations as a tool for understanding ion permeation and selectivity in narrow channels. Biophys. Chem..

[15] Allen T.W., Baştuğ T., Kuyucak S., Chung S.-H. (2003). Gramicidin A channel as a test ground for molecular dynamics force fields. Biophys. J..

[16] Baştuğ T., Chen P.-C., Patra S.M., Kuyucak S. (2008). Potential of mean force calculations of ligand binding to ion channels from Jarzynski’s equality and umbrella sampling. J. Chem. Phys..

[17] Baştuğ T., Kuyucak S. (2007). Application of Jarzynski’s equality in simple *versus* complex systems. Chem. Phys. Lett..

[18] Bhattacharyya S., Ma S., Stankovich M.T., Truhlar D.G., Gao J. (2005). Potential of mean force calculation for the proton and hydride transfer reactions catalyzed by medium-chain acyl-CoA dehydrogenase: Effect of mutations on enzyme catalysis. Biochemistry.

[19] Chen P.-C., Kuyucak S. (2011). Accurate determination of the binding free energy for KcsA-charybdotoxin complex from the potential of mean force calculations with restraints. Biophys. J..

[20] Ding F., Tsao D., Nie H., Dokholyan N.V. (2008). Ab initio folding of proteins with all-atom discrete molecular dynamics. Structure.

[21] Doudou S., Burton N.A., Henchman R.H. (2009). Standard free energy of binding from a one-dimensional potential of mean force. J. Chem. Theory Comput..

[22] Forney M.W., Janosi L., Kosztin I. (2008). Calculating free-energy profiles in biomolecular systems from fast nonequilibrium processes. Phys. Rev. E.

[23] Huang X., Zhao X., Zheng F., Zhan C.-G. (2012). Cocaine esterase–cocaine binding process and the free energy profiles by molecular dynamics and potential of mean force simulations. J. Phys. Chem. B.

[24] Jiang W., Luo Y., Maragliano L., Roux B. (2012). Calculation of free energy landscape in multi-dimensions with Hamiltonian-exchange umbrella sampling on petascale supercomputer. J. Chem. Theory Comput..

[25] Kim T., Im W. (2010). Revisiting hydrophobic mismatch with free energy simulation studies of transmembrane helix tilt and rotation. Biophys. J..

[26] Kosztin I., Barz B., Janosi L. (2006). Calculating potentials of mean force and diffusion coefficients from nonequilibrium processes without Jarzynski’s equality. J. Chem. Phys..

[27] Lee J., Im W. (2007). Restraint potential and free energy decomposition formalism for helical tilting. Chem. Phys. Lett..

[28] Lee M.S., Olson M.A. (2006). Calculation of absolute protein-ligand binding affinity using path and endpoint approaches. Biophys. J..

[29] Miao Y., Feixas F., Eun C., McCammon J.A. (2015). Accelerated molecular dynamics simulations of protein folding. J. Comput. Chem..

[30] Okada O., Odai K., Sugimoto T., Ito E. (2012). Molecular dynamics simulations for glutamate-binding and cleft-closing processes of the ligand-binding domain of GluR2. Biophys. Chem..

[31] Pang J., Pu J., Gao J., Truhlar D.G., Allemann R.K. (2006). Hydride transfer reaction catalyzed by hyperthermophilic dihydrofolate reductase is dominated by quantum mechanical tunneling and is promoted by both inter-and intramonomeric correlated motions. J. Am. Chem. Soc..

[32] Raghav N., Chakraborty S., Maiti P.K. (2015). Molecular mechanism of water permeation in a helium impermeable graphene and graphene oxide membrane. Phys. Chem. Chem. Phys..

[33] Roca M., de Maria L., Wodak S.J., Moliner V., Tunon I., Giraldo J. (2008). Coupling of the guanosine glycosidic bond conformation and the ribonucleotide cleavage reaction: Implications for barnase catalysis. Proteins Struct. Funct. Bioinform..

[34] Shimizu S., Chan H.S. (2002). Anti-cooperativity and cooperativity in hydrophobic interactions: Three-body free energy landscapes and comparison with implicit-solvent potential functions for proteins. Proteins Struct. Funct. Bioinform..

[35] Vashisth H., Abrams C.F. (2008). Ligand escape pathways and (un)binding free energy calculations for the hexameric insulin-phenol complex. Biophys. J..

[36] Zeller F., Zacharias M. (2014). Adaptive biasing combined with Hamiltonian replica exchange to improve umbrella sampling free energy simulations. J. Chem. Theory Comput..

[37] Zeller F., Zacharias M. (2014). Efficient calculation of relative binding free energies by umbrella sampling perturbation. J. Comput. Chem..

[38] Zhu Y., Tong M., Liu C., Song C., Wei D., Zhao Q., Tang M. (2014). Molecular dynamics simulations on inclusion complexes for chiral enantiomers with heterocyclic cyclodecapeptide. Comput. Theor. Chem..

[39] Bagai S., Sun C., Tang T. (2012). Potential of mean force of polyethylenimine-mediated DNA attraction. J. Phys. Chem. B.

[40] Banavali N.K., MacKerell A.D. (2002). Free energy and structural pathways of base flipping in a DNA GCGC containing sequence. J. Mol. Biol..

[41] Baştuğ T., Kuyucak S. (2006). Energetics of ion permeation, rejection, binding, and block in gramicidin A from free energy simulations. Biophys. J..

[42] Berneche S., Roux B. (2001). Energetics of ion conduction through the K&plus; channel. Nature.

[43] Gumbart J.C., Roux B., Chipot C. (2012). Standard binding free energies from computer simulations: What is the best strategy?. J. Chem. Theory Comput..

[44] Gumbart J.C., Roux B., Chipot C. (2013). Efficient determination of protein–protein standard binding free energies from first principles. J. Chem. Theory Comput..

[45] Sun H., Li Y., Tian S., Wang J., Hou T. (2014). P-loop conformation governed crizotinib resistance in G2032R-mutated ROS1 tyrosine kinase: Clues from free energy landscape. PLoS Comput. Biol..

[46] Wang J., Deng Y., Roux B. (2006). Absolute binding free energy calculations using molecular dynamics simulations with restraining potentials. Biophys. J..

[47] Woo H.-J., Roux B. (2005). Calculation of absolute protein–ligand binding free energy from computer simulations. Proc. Natl. Acad. Sci. USA.

[48] Zeller F., Zacharias M. (2014). Evaluation of generalized born model accuracy for absolute binding free energy calculations. J. Phys. Chem. B.

[49] Cheng X., Wang H., Grant B., Sine S.M., McCammon J.A. (2006). Targeted molecular dynamics study of C-loop closure and channel gating in nicotinic receptors. PLoS Comput. Biol..

[50] Wang B., Predeus A.V., Burton Z.F., Feig M. (2013). Energetic and structural details of the trigger-loop closing transition in RNA polymerase II. Biophys. J..

[51] Yamashita T., Fujitani H. (2014). On accurate calculation of the potential of mean force between antigen and antibody: A case of the HyHEL-10-hen egg white lysozyme system. Chem. Phys. Lett..

[52] Banavali N.K., Roux B. (2005). Free energy landscape of A-DNA to B-DNA conversion in aqueous solution. J. Am. Chem. Soc..

[53] Lee J., Kim Y.-G., Kim K.K., Seok C. (2010). Transition between B-DNA and Z-DNA: Free energy landscape for the B–Z junction propagation. J. Phys. Chem. B.

[54] Noy A., Perez A., Laughton C.A., Orozco M. (2007). Theoretical study of large conformational transitions in DNA: The B↔A conformational change in water and ethanol/water. Nucleic Acids Res..

[55] Huang X., Pan Y., Zheng F., Zhan C.-G. (2010). Reaction pathway and free energy profile for prechemical reaction step of human butyrylcholinesterase-catalyzed hydrolysis of (−)-cocaineby combined targeted molecular dynamics and potential of mean force simulations. J. Phys. Chem. B.

[56] Yu T., Schatz G.C. (2013). Free energy profile and mechanism of self-assembly of peptide amphiphiles based on a collective assembly coordinate. J. Phys. Chem. B.

[57] Tong M., Wang Q., Wang Y., Chen G. (2015). Structures and energies of the transition between two conformations of the alternate frame folding calbindin-D_9k_ protein: A theoretical study. RSC Adv..

[58] Chiu T.K., Kubelka J., Herbst-Irmer R., Eaton W.A., Hofrichter J., Davies D.R. (2005). High-resolution X-ray crystal structures of the villin headpiece subdomain, an ultrafast folding protein. Proc. Natl. Acad. Sci. USA.

[59] Kubelka J., Henry E.R., Cellmer T., Hofrichter J., Eaton W.A. (2008). Chemical, physical, and theoretical kinetics of an ultrafast folding protein. Proc. Natl. Acad. Sci. USA.

[60] Ditchfield R., Hehre W.J., Pople J.A. (1971). Self-consistent molecular-orbital methods. IX. An extended gaussian-type basis for molecular-orbital studies of organic molecules. J. Chem. Phys..

[61] Sousa J., Silva P., Machado A., Reis M., Romanielo L., Hori C. (2013). Application of computational chemistry methods to obtain thermodynamic data for hydrogen production from liquefied petroleum gas. Braz. J. Chem. Eng..

[62] Frisch M., Trucks G., Schlegel H., Scuseria G., Robb M., Cheeseman J., Scalmani G., Barone V., Mennucci B., Petersson G. (2009). Gaussian 09, Revision A. 02.

[63] Habenschuss A., Narten A. (1989). X-ray diffraction study of liquid *n*-butane at 140 and 267 K. J. Chem. Phys..

[64] Herrebout W., van der Veken B., Wang A., Durig J. (1995). Enthalpy difference between conformers of *n*-butane and the potential function governing conformational interchange. J. Phys. Chem..

[65] Ota N., Brünger A.T. (1998). Overcoming barriers in macromolecular simulations: Non-Boltzmann thermodynamic integration. Theor. Chem. Acc..

[66] Case D., Darden T., Cheatham T., Simmerling C., Wang J., Duke R., Luo R., Merz K., Pearlman D., Crowley M. (2006). AMBER 9.

[67] Potential of Mean Force (PMF) Calculations with AMBER and WHAM. https://sites.google.com/site/wangtingpage/home/tutorials/pmf.

[68] Murcko M.A., Castejon H., Wiberg K.B. (1996). Carbon–carbon rotational barriers in butane, 1-butene, and 1,3-butadiene. J. Phys. Chem..

[69] Kubelka J., Eaton W.A., Hofrichter J. (2003). Experimental tests of villin subdomain folding simulations. J. Mol. Biol..

[70] Lei H., Wu C., Liu H., Duan Y. (2007). Folding free-energy landscape of villin headpiece subdomain from molecular dynamics simulations. Proc. Natl. Acad. Sci. USA.

[71] Lindorff-Larsen K., Piana S., Dror R.O., Shaw D.E. (2011). How fast-folding proteins fold. Science.

[72] Cellmer T., Henry E.R., Hofrichter J., Eaton W.A. (2008). Measuring internal friction of an ultrafast-folding protein. Proc. Natl. Acad. Sci. USA.

[73] Godoy-Ruiz R., Henry E.R., Kubelka J., Hofrichter J., Muñoz V., Sanchez-Ruiz J.M., Eaton W.A. (2008). Estimating free-energy barrier heights for an ultrafast folding protein from calorimetric and kinetic data. J. Phys. Chem. B.

[74] Kubelka J., Chiu T.K., Davies D.R., Eaton W.A., Hofrichter J. (2006). Sub-microsecond protein folding. J. Mol. Biol..

[75] Duan Y., Wu C., Chowdhury S., Lee M.C., Xiong G., Zhang W., Yang R., Cieplak P., Luo R., Lee T. (2003). A point-charge force field for molecular mechanics simulations of proteins based on condensed-phase quantum mechanical calculations. J. Comput. Chem..

[76] Lee M.C., Duan Y. (2004). Distinguish protein decoys by using a scoring function based on a new AMBER force field, short molecular dynamics simulations, and the generalized born solvent model. Proteins Struct. Funct. Bioinform..

[77] Wang J., Wolf R.M., Caldwell J.W., Kollman P.A., Case D.A. (2004). Development and testing of a general amber force field. J. Comput. Chem..

[78] Schlitter J., Engels M., Krüger P. (1994). Targeted molecular dynamics: A new approach for searching pathways of conformational transitions. J. Mol. Graph..

[79] Schlitter J., Engels M., Krüger P., Jacoby E., Wollmer A. (1993). Targeted molecular dynamics simulation of conformational change-application to the T↔R transition in insulin. Mol. Simul..

[80] Chipot C., Jaffe R., Maigret B., Pearlman D.A., Kollman P.A. (1996). Benzene dimer: A good model for π–π interactions in proteins? A comparison between the benzene and the toluene dimers in the gas phase and in an aqueous solution. J. Am. Chem. Soc..

[81] Kumar S., Bouzida D., Swendsen R.H., Kollman P.A., Rosenberg J.M. (1992). The weighted histogram analysis method for free-energy calculations on biomolecules. I: The method. J. Comput. Chem..

[82] Patey G., Valleau J. (1975). A Monte Carlo method for obtaining the interionic potential of mean force in ionic solution. J. Chem. Phys..

[83] Torrie G.M., Valleau J.P. (1977). Nonphysical sampling distributions in Monte Carlo free-energy estimation: Umbrella sampling. J. Comput. Phys..

[84] Weighted Histogram Analysis Method for Analyzing Umbrella Sampling Simulation Data. http://membrane.urmc.rochester.edu/content/wham.

[85] Interhelical Angle Program (with Sign Designation). http://nmr.uhnres.utoronto.ca/ikura/resources/data+sw/interhlx.

[86] Sadiq S.K., de Fabritiis G. (2010). Explicit solvent dynamics and energetics of HIV-1 protease flap opening and closing. Proteins Struct. Funct. Bioinform..

[87] Miyamoto S., Kollman P.A. (1992). SETTLE: An analytical version of the SHAKE and RATTLE algorithm for rigid water models. J. Comput. Chem..

[88] Yap K.L., Ames J.B., Swindells M.B., Ikura M. (1999). Diversity of conformational states and changes within the EF-hand protein superfamily. Proteins Struct. Funct. Bioinform..

