# Supplementary material for: Study on the Application of the Combination of TMD Simulation and Umbrella Sampling in PMF Calculation for Molecular Conformational Transitions
Source: Int J Mol Sci. 2016 May 9;17(5):692. doi: 10.3390/ijms17050692 (PMC4881518; doi:10.3390/ijms17050692)
Supplement: Supplementary file 1 [file ijms-17-00692-s001.pdf]

Supplementary Materials: Study on the Application of the Combination of TMD Simulation and Umbrella Sampling in PMF Calculation for Molecular Conformational Transitions

Qing Wang, Tuo Xue, Chunnian Song, Yan Wang and Guangju Chen

1. Conventional Molecular Dynamics Simulation Protocols

All conventional molecular dynamics (CMD) simulations for these built models were carried out using the AMBER 9 package [66] and ff03 all atom force field parameters [75–77]. The protocol for all CMD simulation was described as follows: (1) the systems were energetically minimized to remove unfavorable contacts. Four cycles of minimizations were performed with 2500 steps of each minimization and harmonic restraints on a butane molecule and the HP35 protein from 100, 75, 50 to 25 kcal/(mol·Å^2), which means that the restraints were relaxed stepwise by 25 kcal/(mol·Å^2) per cycle. The fifth cycle consists of 5000 steps of unrestrained minimization before the heating process. The cutoff distance used for the non-bonded interactions was 10 Å. The SHAKE algorithm [87] was used to restrain the bonds containing hydrogen atoms; (2) each energy-minimized structure was heated over 120 ps from 0 to 300 K for butane and to 340 K for the HP35 protein (with a temperature coupling of 0.2 ps, while the positions of the molecules were restrained with a small value of 25 kcal/(mol·Å^2). The constant volume was maintained during the processes; (3) the unrestrained equilibration of 200 ps with constant pressure and temperature conditions was carried out for each system. The temperature and pressure were allowed to fluctuate around 300/340 K and 1 bar, respectively, with the corresponding coupling of 0.2 ps. The Langevin thermostat (NTT = 3) was used for the temperature regulation of each system. For each simulation, an integration step of 2 fs was used; and (4) finally, CMD runs of 6 ns for the HP35-R model were carried out, respectively, by following the same protocol.

2. Calculations of Interhelical Angle, Hydrogen Bond and Correlation of Atomic Motions

In the calculations of interhelical angle, the program INTERHLX calculates the sign of the angle between two helices by following this convenient role: the two helices are taken to be positioned by helix I being in front of helix II. Helix I (from N to C) is used to define first vertical vector. A second vertical vector is defined with its tail at the C-terminus of helix II. The angle between helices I and II is the rotation required to align the head of the second vector with the N-terminus of helix II. The vector is rotated in the direction that produces an angle of less than 180 degrees with the clockwise or counterclockwise rotation represented by positive or negative sign. This program can also provide other geometry based parameters such as interhelical distances [88].

To examine the unfolding process of the HP35 protein, the occupancies of all possible intra-helix hydrogen bonds at the three helices of the protein were measured by calculating the percentage of snapshots during the simulation that the hydrogen bonds existed. A "hydrogen bond" was defined as a distance of less than 3.5 Å between a hydrogen atom attached to either an oxygen or a nitrogen atom and an acceptor atom, and as an angle formed by a donor, a hydrogen atom and an acceptor being larger than 120° with the corresponding occupancy of ≥50%. To compare the amount of the total possible hydrogen bonds in the various transition conformations, a relative percentage of total hydrogen bond occupancies for a conformation was calculated by defining the relative percentage equal to one hundred percent when the all possible hydrogen bonds occupy each snapshot in the reactant during the HP35 unfolding simulation.

The correlation coefficients were averaged over the regions of protein, and the resulted cross-correlation coefficients are presented in the form of a two-dimensional graph [86]. These structure analyses in the present work were calculated by using the PTRAJ module of the AMBER 9 program [66].

Table S1. Optimized structural parameters for four C atoms in butane molecule along with experimental data (bond: angstrom and angle: degree).

Parametes	Optimization	Experiment
**C1–C2**	1.534	1.550 [a] (1.533) [b]
**C1–C3**	2.564	2.559 [a] (2.547) [b]
***g* C1–C4**	3.191	3.1 [a] (3.072) [b]
***t* C1–C4**	3.937	3.900 [b]
**C–C–C**	113.36	112.7 [c]
**C–C–C–C**	180	180 [d]

[a,b] Taken from Ref. [63]; [c] Taken from Ref. [65]; [d] Taken from Ref. [64]; *g* represents *gauche*-like butane; *t* represents *trans*-like butane.

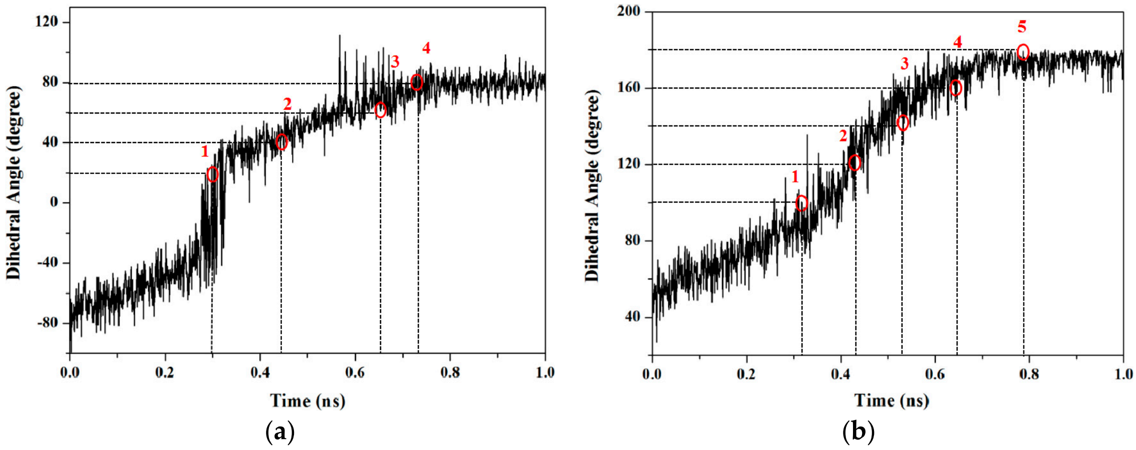

Figure S1. The dihedral angle values of C–C–C–C and the selected sampling windows of (**a**) 4 for the second TMD simulation; and (**b**) 5 for the third TMD simulation. The selected sampling windows are numbered around the red circles. The corresponding expected values and snapshots with the red circles are marked by the dash lines.

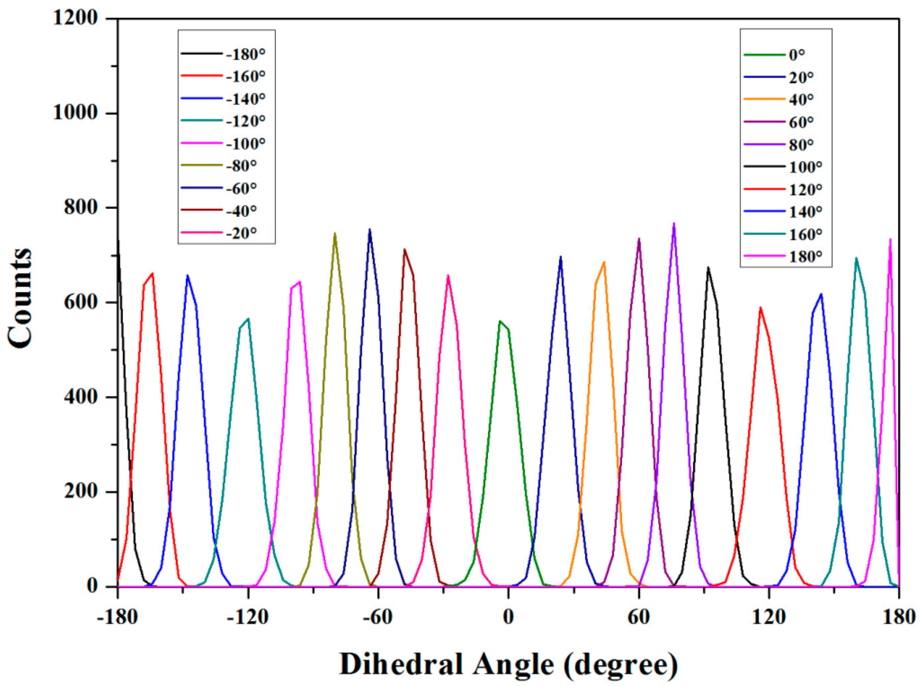

Figure S2. The checked histograms of the dihedral angles from 19 umbrella sampling simulation trajectories for the rotational transition of butane from −180° to 180°.

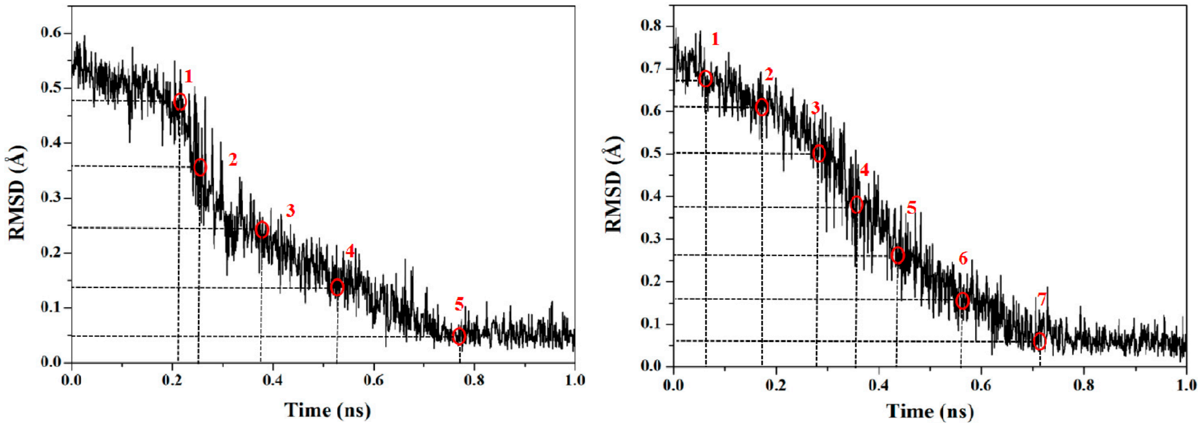

(**a**) (**b**)

Figure S3. The root mean square deviation (RMSD) values of four C atoms and the selected sampling windows of (**a**) 5 for the second TMD simulation; and (**b**) 7 for the third TMD simulation. The selected sampling windows are numbered around the red circles. The corresponding expected values and snapshots with the red circles are marked by the dash lines.

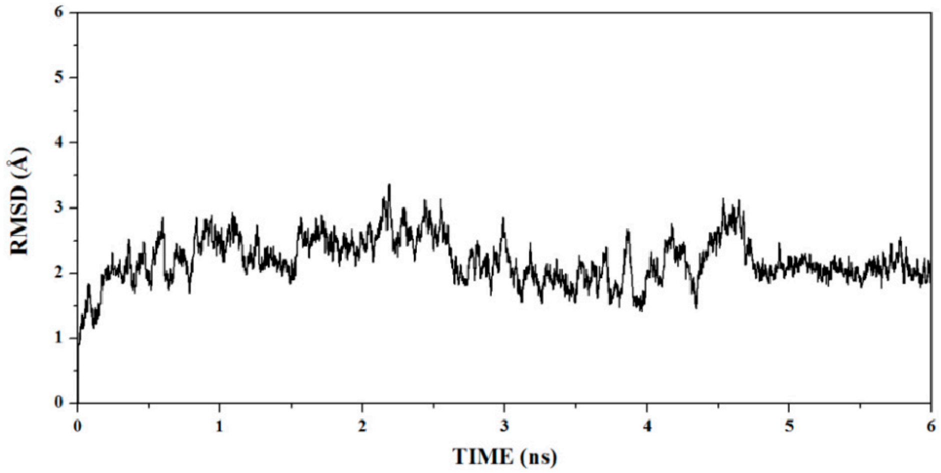

Figure S4. Root mean square deviation (RMSD) values of all backbone atoms of HP35 protein from its simulation at 340 K with respect to the corresponding starting structure.
